# SNP marker development in *Pinus sylvestris* L. in stress-responsive genes characterized from *Pinus cembra* L. transcriptomes

**DOI:** 10.1007/s11033-020-05527-y

**Published:** 2020-05-19

**Authors:** Zoltán A. Köbölkuti, Endre Gy. Tóth, Daniela Jahn, Berthold Heinze, Mária Höhn

**Affiliations:** 1 Department of Botany, Faculty of Horticulture, Szent István University, Ménesi út 44, Budapest, 1118 Hungary; 2 Department of Breeding, National Agricultural Research and Innovation Centre, Forest Research Institute, Várkerület 30/A, Sárvár, 9600 Hungary; 3 Forest Research Institute (IRF), University of Quebec in Abitibi-Témiscamingue UQAT, 445 Boul. de l’Université, Rouyn-Noranda, QC J9X 5E4 Canada; 4 Federal Research and Training Centre for Forests, Natural Hazards and Landscape (BFW), Seckendorff-Gudent-Weg 8, 1130 Vienna, Austria

**Keywords:** *Pinus cembra*, *Pinus sylvestris*, Adaptation, Candidate genes, SNP markers

## Abstract

Massively parallel sequencing of cDNA is an efficient route for generating sequence collections that represent expressed genes under different environmental control. The analysis of their sequence helps in developing molecular markers, such as SNPs, which represent a useful tool in detecting adaptive signals in populations. In this study novel PCR markers, based on stress responsive genes, were designed from the transcriptome of the haploxylon Swiss stone pine (*Pinus cembra* L.) and tested for SNPs in the diploxylon Scots pine (*Pinus sylvestris* L.). 84 primers were tested on *P. sylvestris* DNA samples originating from three different types of habitat. After sequencing and BLAST search of the amplified products, parts of 19 different candidate genes were analysed by considering the polymorphic sites, insertions/deletions as well as synonymous and non-synonymous SNPs. In a total of 3735 sites no indels, eight synonymous and 11 non-synonymous SNPs were found. By providing de novo molecular markers developed in *P. cembra* and tested for transferability in Scots pine, our results give support for the use of de novo markers targeting conserved regions across different pines. The SNPs detected may have important applications in further studies of adaptive genetic variation, providing tools to study relevant genes important in the long-term adaptation of pine species.

**Electronic supplementary material:**

The online version of this article (10.1007/s11033-020-05527-y) contains supplementary material, which is available to authorized users.

## Introduction

Focus on plant response to environmental stress is becoming increasingly important, as most future climate scenarios suggest climate change that imply an increase in aridity of many areas, causing abiotic stress and seriously threatening natural ecosystems [1]. Aridity influences drought-response trait differentiation and genetic divergence among populations. In these conditions, taking into consideration the genetic information related to adaptation is fundamental in developing conservation strategies [2].

An important way to obtain genetic information is by genome sequencing. Nevertheless, in case of conifers, due to the large genomes, only a few species have been sequenced, so far. This is an obstacle to the genetic evaluation of a large number of species. As an alternative, massively parallel transcriptome sequencing is an efficient route to develop genetic markers [3], which can be used in genetic analysis of different conifer species [4].

Haploxylon Swiss stone pine (*Pinus cembra* L.), a soft pine, which has one fibrovascular bundle and diploxylon Scots pine (*Pinus sylvestris* L.), a hard pine, which has two, both belonging to subgenus *Pinus* but belonging to different subsections namely *Strobus* and *Pinus* and the latter tending to have harder timber and a larger amount of resin, have formerly been described as highly sensitive to climatic changes [5, 6]. Scots pine, as a widely tolerant pioneer species, surviving close to the permafrost during the Pleistocene has adapted to different climates, being able to colonize even recently man-disturbed areas [7]. Evaluating genetic variation of the species with focus on the stress-adaptive genes by appropriate genetic tools could provide useful information for the conservation of native remnant populations as a biodiversity resource for the future.

The study was performed to describe (1) homologues in the *P. cembra* transcriptome to formerly annotated stress responsive genes, (2) test on the applicability of primers designed on these gene fragments in *P. sylvestris*, and (3) genotype by PCR and Sanger sequencing Scots pine samples of different habitat types by revealing possible nucleotide variation at these loci.

## Material and methods

### Plant material

*Pinus cembra* samples were from a previous comparative study (European larch and Swiss stone pine) (Jahn and Heinze, unpublished), for which material was sampled from six sites along the Austrian Alps (Table S1), 15 individuals from each population. From every tree 2–5 two-year-old brachyblasts with healthy needles and female cones were collected at four collection stages (June, July, August and September 2015), and male flowers at two stages, in June and July. For RNA extraction, tissues were stored in liquid nitrogen. Among all samples, RNA seq was initially performed on two cones in different developmental stages (collected in June and Sept respectively) and a needle, all three samples originating from one tree in Obergurgel (°N 46.86; °E 11.01) This transcriptome (as described below) was used in the present study. The primers designed on de novo identified gene sequences were tested in the laboratory on 84 *P. cembra* DNA samples, selected at random, all from the collected needles from the six sites previously mentioned. Scots pine samples (10–20 2-year-old brachyblasts with needles/one individual/population) originated from the Carpathian area, from three natural populations formerly included in a microsatellite study [8] (for details see Table S2), originating from three different types of habitat: wet mountain raised bog from the Eastern Carpathians [Mohos (RO)]; dry rocky outcrop of the lower Tatra region [Kvacany (SK)] and beech-pine mixed forest from the prealpine region of Western Hungary [Csörötnek (HU)], DNA being extracted from one sample/population/habitat type.

### De novo identified gene sequences in *P. cembra*

**Fig. 1:**
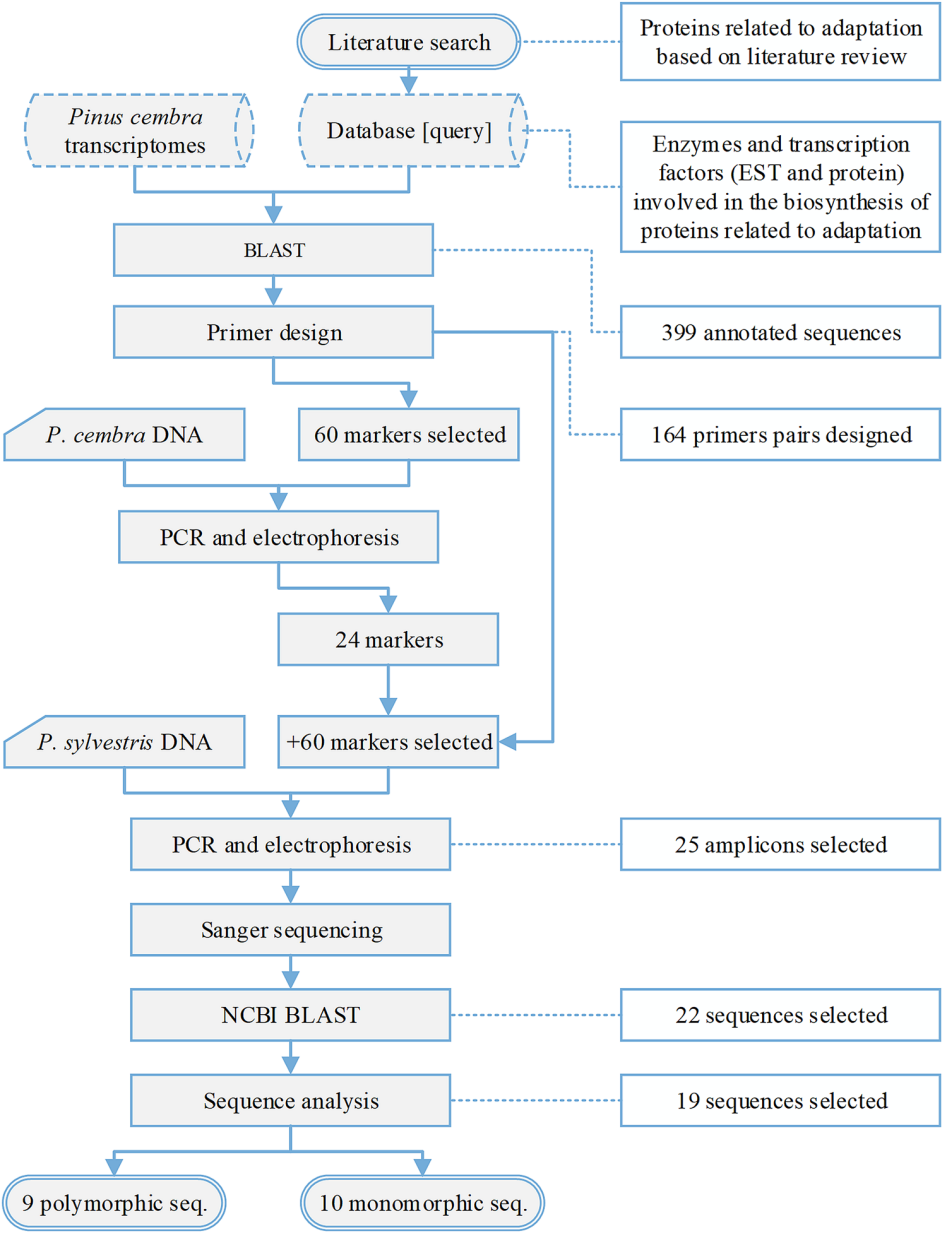
Diagrammatic representation of the most important steps of gene annotation, primer design and PCR test in *P. cembra*, transfer and sequence analyses during marker development in *P. sylvestris*

The most important steps of primer design, testing, sequencing, and analyses of the amplified sequences we highlighted in a flowchart (Fig. 1). RNA was extracted from diploid tissues (scales and needles) by using MasterPure Plant RNA purification Kit (Invitrogen, Epicentre, USA), using the manufacturer’s protocol. Messenger RNA was isolated with Dynabeads mRNA DIRECT Micro Kit (ThermoFisher Scientific, Carlsbad, CA, USA) using the manufacturer’s protocol, followed by an evaluation according to their RIN value (Bioanalyzer, Agilent, Santa Clara, CA, USA).

Extracted mRNA was used to amplify cDNA libraries, according to the Ion Torrent RNA-Seq protocol (ThermoFisher Scientific, Carlsbad, CA, USA). The RNA Seq analysis was done using an Ion Torrent platform. For each sample a single library was prepared using all reagents and protocols for the Ion Torrent™ Personal Genome Machine™ (PGM) System. The quality and quantity of each preparation step was evidenced using a Bioanalyzer Instrument and each library was loaded on a 316 Ion Torrent Chip. After sequencing, further analysis was done using the CLC Bio Genomics Workbench. The output of the library prepared from female cone in the early developmental stage (June) contained 2.1 million reads with a mean read length of 88 bp and a total of 194 Mbp. The library created from needles gave a total of 257 Mbp, a mean read length of 90 bp and a read count of 2.7 million reads, while the library from the more developed female cone (July) resulted in a mean read length of 83 bp, a total of 147 Mbp and around 2.1 million reads. At first, raw reads were quality-trimmed using a quality threshold of 20. Thereafter, from the library of the female early cone, simple contig sequences were created with a minimum aligned read length of 50 bp, medium alignment stringency, word size 20, bubble size 50. The de novo assembly of this library resulted in 43,000 contigs with a mean length of 1300 bp. Those were used as internal reference in RNA-Seq mapping for the other libraries.

In parallel, data mining was performed, from literature where candidate proteins (enzymes and transcription factors) with possible roles in adaptation were selected. Table S3 presents the candidate proteins selected, based on literature, the presumable coding sequences of which were annotated and PCR cloned during the study.

Based on Table S3, a BLAST database was filled with sequences downloaded from NCBI database (https://www.ncbi.nlm.nih.gov, 07.12.2016), using the following criteria: first, conducting a search for EST and protein sequences for these enzymes and transcription factors that were annotated in the *Pinus* genus. If such could not be found, the search was extended to ‘‘Pinaceae”, and if unsuccessful, to ‘‘land plants”. The contents of the BLAST database are presented in Table S4.

De novo assembled contigs were searched using BLASTN and TBLASTN toolkits against the database with CLC Genomic Workbench version 9.0 (QIAGEN Bioinformatics). After the search based on sequence homology, the putative function of the sequences was assigned according to the highest BLAST hits. By this method, from the transcriptome, sequences were selected, annotated and renamed by the coded protein. All sequences were searched again with the BLAST Genome function of the NCBI (https://blast.ncbi.nlm.nih.gov/Blast.cgi), against the *Pinus lambertiana* genome (taxid:3343) by BLASTN, optimized for somewhat similar sequences, with algorithm parameters: max target sequences 100, expect threshold 10, word size 11, max matches in a query range 0, match/mismatch scores gap costs 2–3 and filtering the low complexity regions and by using a species-specific repeats filter for sugar pine. *Pinus lambertiana* is the closest sequenced relative of *P. cembra*. This search should indicate any possible introns, as we designed the primers for use with genomic DNA. Primers were designed only on sequences that showed a high degree of similarity and equality in length to translated regions in the *P. lambertiana* genome according to the highest BLAST hits. Following this step, a smaller number of suitable sequences were selected for primer design. The list of the designed primer pairs is presented in Table S5. The design of the primers was performed with the primer design toolkit of CLC Genomic Workbench.

To test the amplification, from the newly designed primers, 60 with the most favorable primer selection parameters were selected and tested in the laboratory on 84 randomly selected Swiss stone pine DNA samples (from needles of the six sites previously mentioned in the “Plant material” section), as follows: in a 14 µl volume containing 1 µl of genomic DNA (about 20 ng), 0.14 µl Polim Phite Taq, GeneAmp PCR buffer II (Applied Biosysytems/Roche, Branchburg, N.J.), 3 mM MgCl_2_, 200 μM of dNTPs, 0.2 mM of each primers, by the following PCR (SPECPCR) protocol: denaturation on 94 °C for 3 min (1); 60 °C for 1 min (2), 70 °C for 1 min (3); 9 × up to step 3 (4); 94 °C for 30 s (5); low stringency annealing 55 °C for 50 s (6); 70 °C for 2 min (7); 34 cycles up to step 7 (8).

The PCR products were not genotyped by sequencing, being analysed only visually concerning their presence or absence, number and size, by 2% agarose gel electrophoresis with 3:1 Biogyn Sieve agarose: normal agarose and 1 × TAE as electrophoresis buffer.

### Test of the designed primers in Scots pine

DNA extraction from Scots pine samples was done from 20 to 25 mg of plant material of 2-year old needles by using DNeasy Plant Mini Kit (QIAGEN, Valencia, CA, USA) according to the manufacturer’s protocol. For testing the newly designed primers, a preliminary PCR test was carried out to evaluate their functionality and transferability. From all primers designed, those that previously amplified only one target in *P. cembra* and another 60 with optimal primer selection parameters (in total 84 primer pairs) were selected to check for the amplification in Scots pine samples. PCR amplification for this purpose was conducted in a 15 µl volume containing 1 µl of genomic DNA (about 20 ng), 0.14 µl Polim Phite Taq (1 unit) and, GeneAmp PCR buffer II (Applied Biosysytems/Roche, Branchburg, N.J.), 3 mM MgCl_2_, 200 μM of dNTPs, 0.2 mM of each primer, following the PCR protocol: denaturation (1) at 94 °C for 3 min; followed by (2) 94 °C for 30 s; annealing (3) at 55 °C for 0.45 s and extension (4) at 72 °C for 1.20 min, (5) 72 °C for 10 min; the first four cycles being repeated 34 times. The PCR products were analysed by 1% agarose gel electrophoresis with 1 × TAE as electrophoresis buffer. Further, only the products that appeared as a single band similar to the expected size (in *P. cembra*) were used to genotype by Sanger sequencing samples with different origin.

For sequencing, the purification of the products after PCR was done by hydrolyzing the excess primers and dephosphorylated unincorporated dNTPs, in one step, with CleanSweep PCR purification reagent (ThermoFisher Scientific, Carlsbad, CA, USA), according to the manufacturer’s protocol. The products resulting from the CleanSweep process were then sequenced using the forward primers, in one direction, at Biomi Ltd., Hungary and at the Institute of Genetics, Biological Research Centre of the Hungarian Academy of Sciences, Szeged, Hungary.

Editing, visualization and alignment of the amplified sequences was accomplished with BioEdit Sequence Alignment Editor version 7.0.9.0 [9] software. Following sequence alignment, genotyping and final validation of all potential SNPs was made visually on chromatograms with use of CodonCode Aligner 8.0.2 (CodonCode Corporation). Number of polymorphic sites, number of insertions/deletions and the character of SNPs (synonymous or non-synonymous) was calculated using DNA Sequence Polymorphism v6.10.01 (DNASP) software [10] and validated after a visual check in CodonCode Aligner 8.0.2. Number of synonymous and non-synonymous sites were computed as Nei and Gojobori [11], excluding all cases that go through stop codons. Since our sampling design (one diploid sample/population) did not make possible the calculation of excess heterozygosity, and our marker development started from *P. cembra* transcriptomes as well, the paralogous state of our sequences was tested by homology-based search with the list of single- and multi-copy contigs of Rellstab et al. [12], available at Dyriad digital repository: 10.5061/dryad.4bb5849 and the *P. sylvestris* genome draft [13] with Galaxy (https://usegalaxy.org/) platform’s NCBI BLAST + blastn tool (expectation value cutoff 0.001; filtering criteria: the same alignment length as the length of the query) and by transposon search with LTR_FINDER (min LTR length 25, max LTR length 3500, output score threshold 6.0, predicting PBS by using *Arabidopsis thaliana* L. tRNA database) [14].

## Results and discussion

### Screening the *P. cembra* transcriptome data for sequence homologs and testing the markers on *P. cembra* DNA

Contigs assembled from the three *P. cembra* transcriptomes were screened using BLASTN and TBLASTN toolkits against the BLAST database. From all, 399 sequences were selected, annotated and renamed by the coded protein. The tested 60 primers successfully amplified 42 sequences on 84 different *P. cembra* DNA samples, and 24 PCR products appeared as a single band in the electrophoresis gel (for visual example of three particular amplicons, see Fig S1). Further investigation of these fragments could be a part of a different study concentrating on *P. cembra*, as we found out that they are not present in Rellstab et al.’s lists [12], as detailed below.

### Testing the amplified sequences in *P. sylvestris*

From all primers producing one band amplicons in Swiss stone pine, complemented as described in the “Material and methods” section and tested in *P. sylvestris* DNA samples, 53 provided PCR products. 25 PCR products out of these appeared as a single band in the gel (Fig S2). Our study is a PCR primer transferability-SNP discovery test and according to literature, SNP markers from transcriptome-derived sequences already have been developed and utilized in diverse conifer species [4, 15, 16].

After sequencing, good quality sequences were obtained in case of 22 sequences. A number of three sequences (NAD + 4472, F'3H2550, CHS7594) showed no clear chromatograms, being excluded from further analyses, and CHS381 and MADS1818 showed clear sequencing chromatograms only for PCR products of two samples each. After the homology-based search by BLASTN and BLASTX against the NCBI Genomic Reference Sequences and the NCBI Non-redundant protein seq Database, respectively, in case of three (APX8272, APX637, SOD4683) no significant similarity was found. Accordingly, these sequences were also excluded from further analyses. Table S6 summarizes the results of the BLASTN search against the NCBI Genomic Reference Sequences.

Our results suggest that such primers could work across a wider range of *Pinus* species. 25 out of 84 primers amplified genomic regions of the expected size, presumably for the reason that these sequences are in interspecies conserved regions of the genus. These type of tests in conifers were reported in several studies like in Hansen et al. [17], Kormutak [18], Chen et al. [19], Liewlaksaneeyanawin et al. [20], Lesser et al. [21] or in Sakaguchi et al. [22].

Our rate of success of transferability was 29.76%, which is lower than that of EST-SSR markers from *Pinus taeda* to *Pinus elliottii* var. elliottii (Engelm.) and *Pinus caribaea* var. hondurensis (Sénécl.) (58%) [23]. However, taking into consideration that *P. cembra* and *P. sylvestris* are more distant species compared to *P. elliottii* var. elliottii and *P. caribaea* var. hondurensis, this result could be expected.

The results of the sequence analysis of 19 sequences with evaluable chromatograms (in total 4037 sites) using ClustalW and DNA Sequence Polymorphism v6.10.01 are summarized in Table 1. In total, 21 SNPs were found, of which seven alone in Myb 4095. In enzyme coding genes, the number of SNPs was 14 and ranged from one to five. In a second phase of the analysis, the number of indels and the character of SNPs (synonymous or non-synonymous) was determined, the results being summed in Table 1.

**Table 1:** Outputs of the sequence analysis in *P. sylvestris* samples with DnaSP v.5.0 effectuated on 55 PCR products amplified by 19 primers

Abbrev.	Nr. of sites	Nr. of polymorphic sites	No. of non-synonymous SNPs	No. of synonymous SNPs
Myb 4633	297	0	0	0
Myb 4095	300	7	6	1
WRKY9928	176	0	0	0
WRKY20368	207	0	0	0
WRKY4214	183	0	0	0
WRKY1289	321	0	0	0
MADS15369	166	1	1	0
MADS1818*	300	0	0	0
MADS2038	144	0	0	0
MADS12384	188	1	1	0
PAL1134	177	3	1	2
CHS381*	301	2	2	0
CHS4014	178	0	0	0
F3H350	202	1	0	1
IGSTP657	263	5	3	2
diTPS11241	133	0	0	0
diTPS5871	147	0	0	0
APX802	277	3	2	1
SOD3685	77	2	1	1
Total	4037	21	11	8

Genes for which only two sequences were analysed are marked with *

Out of 19 sequences, no indels were found. One or two synonymous single nucleotide polymorphisms were found in case of six sequences. Non-synonymous single base mutations ranged from one to six, in case of eight of the analysed sequences. Despite the conserved nature of the primer binding sites, SNP polymorphisms do not seem to be conserved between the samples from the different Scots pine habitat types. This can be tested in a follow-up study.

As a step prior to investigating the polymorphism detected in sequences it is necessary to consider that genes could be pseudogenes, which might lead to incorrect results and false interpretations. Formed by chromosomal duplications or transpositions, these are important features of multi-gene families of large eukaryotic genomes, especially of conifers [24]. By testing the paralogous state of our sequences by homology-based search with the list of single- and multi-copy contigs of Rellstab et al. [11], interestingly, none of our 19 queries were found either among all their released sequences. In our consideration, this fact may be caused by the different sampling design. Two of the specific tissue types (early and mid-stage developing cones) that were used in our study were probably not present among the tissue types of Rellstab et al. [12], who used ‘‘male and female cones of an adult tree” without specifying their developmental stages in the description. Furthermore, authors used many more tissues and their tree at the Institute location (around Zürich, Switzerland) at unusually low elevation for this species compared to the Obergurgl site at around 2000 m above sea level in our study may result in different gene expression patterns.

We continued our search by BLASTN against the assembled *P. sylvestris* genome. All queries could be aligned to the draft genome, one candidate (CHS4014) being present at the same alignment length, in two different contigs. Last, we searched our sequences looking for transposable elements using LTR_FINDER. Since no transposons were found and our BLAST results indicated sequence specificity, with only one exception (CHS4014), we conclude that the majority of our designed markers are not pseudogenes.

As variation in relevant genes is essential for the long-term adaptation process, in our consideration, several markers developed here should give interesting new insight in terms of polymorphism. Those that proved to be polymorphic in *P. sylvestris* samples from different habitats, could be used in prospective studies, with large number of individuals, to reveale adaptive divergence between distinct habitat types.

## Electronic supplementary material

Below is the link to the electronic supplementary material.

Supplementary file1 (DOCX 1771 kb)
