# Supplementary material for: SNP marker development in Pinus sylvestris L. in stress-responsive genes characterized from Pinus cembra L. transcriptomes
Source: Mol Biol Rep. 2020 May 19;47(6):4841–7. doi: 10.1007/s11033-020-05527-y (PMC7295734; doi:10.1007/s11033-020-05527-y)
Supplement: Supplementary file 1 — Supplementary file1 (DOCX 1771 kb) [file 11033_2020_5527_MOESM1_ESM.docx]

**Supplementary material**

**Stress responsive SNP marker development in *Pinus cembra* L., transferability and characterization in *Pinus sylvestris* L.**

**Zoltán A. Köbölkuti^a,b^, Endre Gy. Tóth, Daniela Jahn, Berthold Heinze, Mária Höhn**

^a^Szent István University, Department of Botany, Faculty of Horticulture,1118, Ménesi út 44, Budapest, Hungary

^b^National Agricultural Research and Innovation Centre, Forest Research Institute, Department of Breeding, 9600, Várkerület 30/A, Sárvár, Hungary

E-mail: [kobolkuti.zoltan@erti.naik.hu](mailto:kobolkuti.zoltan@erti.naik.hu), Phone: +36305631196

**Tables**

Table S1. List of the six populations of *Pinus cembra* from the Austrian Alps included in this study.

| **Residential area** | **Latitude** **(°N)** | **Longitude** **(°E)** | **Samples** |
| --- | --- | --- | --- |
| Obergurgl | 46.86 | 11.01 | 15 |
| Stoderzinken | 47.45 | 13.81 | 15 |
| Oberhauser Zirbenwald | 46.94 | 12.33 | 15 |
| Turracher Höhe | 46.93 | 13.87 | 15 |
| Petzen | 46.51 | 14.75 | 15 |
| Donnersbach | 47.46 | 14.13 | 15 |

Table S2. List of the three populations of *Pinus sylvestris* included in this study. Populations originate from Central and Eastern Europe, the periphery of species’ distribution area.

| **Code** | **Country** | **Region** | **Residential area** | **Latitude (°N)** | **Longitude (°E)** | **Altitude (m a.s.l.)** | **Est. area (km^2^)** | **Samples** | **Comments** |
| --- | --- | --- | --- | --- | --- | --- | --- | --- | --- |
| HOR | Hungary | PB | Csörötnek | 46.93 | 16.35 | 296 | 0.10 | 23 | Mixed forest |
| SKV | Slovakia | WC | Kvacany | 49.18 | 19.54 | 799 | 0.48 | 20 | Rock surface |
| RMO | Romania | EC | Baile Tusnad (Mohos) | 46.13 | 25.91 | 1052 | 0.58 | 29 | Peatbog |
| PB Pannonian Basin, WC Western Carpathians, EC Eastern Carpathians | | | | | | | | | |

Table S3. Selected proteins related to adaptation according to the literature.

| **Name (abbrev.)** | **Protein** | **Role in the adaptation process** | **Reference and investigated species** |
| --- | --- | --- | --- |
| APX | Ascorbate peroxidase | Role in freezing tolerance during cold acclimatization; against high production of toxic oxygen; low light; pollution | [1] *Pinus sylvestris*; [2] *Pinus strobus*; [3] *Pinus taeda*; [4, 5] *Picea asperata*; [6] *Abies mariesii*; [7,8] *Pinus sylvestris* |
| CHI; CHS, F3H, F3’H, F3’5’H, LDR, UDP; ANS | Chalcone synthase; chalcone isomerase; flavanone hydroxylase; flavonoid 3’hydroxylase; flavonoid 3’5’ hydroxylase; leucoanthocyanidin reductase; anthocyanidin synthase; 3-O-glucosyltransferase | Role in responses to abiotic signals, including UV damage, temperature fluctuations and low availability of nutrients and water; fungal infection; | [2]*Larix decidua, Picea abies, Pinus silvestris, P. parviflora, Abies procera, Abies koreana, and Abies concolor*; [9] *Pinus* *sylvestris*; [10] *Picea abies*; [11] *Pinus banksiana* |
| MDH | NAD malate dehydrogenase | Role in responses to adverse environmental conditions, invading organisms and ultraviolet irradiation | [12] *Abies concolor*, *Abies grandis; Picea abies; Picea glauca; Picea mariana; Picea sitchensis; Pinus banksiana; Pinus contorta; Pinus ponderosa; Pinus rigida; Pinus sylvestris; Pinus taeda; Pseudolsuga menziesii; Thuja plicata*; |
| SOD | Superoxide dismutase | Role in response to toxic oxygen species | [13] *Pinus sylvestris, Picea abies*; [14] *Pinus sylvestris* |
| diTPS | Diterpene synthase | Role in response to biotic and abiotic environmental factors | [15] *Pinus strobus*; [16] several conifer species |
| MADS | MADS box proteins | Role in floral development, organ identity | [17-23] *Picea abies, Picea mariana, Pinus radiata, Pinus resinosa* |
| MYB | MYB superfamily | Role in tolerance to freezing, drought and salt stress; plant hormone and pathogen-mediated stress responses; regulation of flavonoid accumulation; in regulation of lignin synthesis enzymes | [24, 25] *Picea glauca, Pinus taeda*; [26] *Picea mariana*; [27] gymnosperms |
| WRKY | WRKY proteins | Role in reprogramming of plant immune responses; regulation of disease resistance | [28] *Pinus elliottii*, *Pinus sylvestris, Pinus radiata, Pinus taeda*; [29] *Pinus monticola* |

Table S4. BLAST database with the downloaded protein and EST sequences involved in plant’s adaptive processes.

| **Abbrev.** | **Name** | **Protein seq** | **EST seq** |
| --- | --- | --- | --- |
| PAL | Phenylalanine ammonia lyase | 790 | 9 |
| CHS | Chalcone synthase | 277 | 25 |
| CHI | Chalcone isomerase | 14 | 260 |
| F3H | Flavanone 3-hydroxylase | 43 | 110 |
| F3'H | Flavanoid 3'-hydroxylase | 56 | 2 |
| F3'5'H | Flavonoid 3'5'-hydroxylase | 235 | 6 |
| DFR | Dihydroflavonol reductase | 26 | 7 |
| LDOX | Leucoanthocyanidin dioxygenase | 15 | 201 |
| LAR | Leucoanthocyanidin reductase | 41 | 34 |
| ANS | Anthocyanidin synthase | 363 | 2 |
| ANR | Anthocyanidin reductase | 26 | 1 |
| 3GT | Flavonoid 3-glucosyltransferase | 3 | 41 |
| AAT | Anthocyanin acyltransferase | 96 | 48 |
| 5GT | Anthocyanin 5-o-glucosyltransferase | 31 | 8 |
| AMT | Anthocyanin methyltransferase | 32 | 19 |
| 3RT | Rhamnosyltransferase | 26 | 2 |
| IGSTP | Glutathione S-transferase | 365 | 31 |
| UFGT | Flavonoid-3-O-glucosyltransferase | 3 | 2 |
| ALT | Alanine aminotransferase | 8 | 17 |
| AST | Aspartate aminotransferase | 36 | 8 |
| NAD+ | NAD malate dehydrogenase | 24 | 29 |
| AroE | Shikimate dehydrogenase | 22 | 65 |
| GPI | Glucose 6 phosphate isomerase | 24 | 18 |
| 6PGD | 6 phosphogluconate dehydrog. | 15 | 29 |
| RuBisCo | Ribulose-1,5-bisphosph. carboxyl./oxyg. | 8 | 65 |
| diTP | Diterpene synthase | 15 | 58 |
| SUS | Sucrose synthase | 29 | 34 |
| SOD | Superoxide dismutase | 26 | 32 |
| APX | Ascorbate peroxidase | 50 | 67 |
| PFK | Phosphofructokinase | 49 | 23 |
| AcI | Acid invertase | 36 | 76 |
| GR | Glutathione reductase | 8 | 73 |
| MADS | MADS-box | 11 | 3 |
| WRKY | WRKY-transcription factor | 555 | 163 |
| Myb | Myb-transcription factor | 9 | 252 |

Table S5. List of the designed 164 primers (with each pairs’ nucleotide succession and length of the amplified sequence).

| **Code** | **Sequence Fwd** | **Sequence Rev** | **Length of the amplified fragment** |
| --- | --- | --- | --- |
| Myb1372 | CTGATTGCAGGGCGTATT | CTTCAACATCATTGCGTCC | 172 |
| Myb4633 | AAGCAGCAGCCAACAAAA | GCAAAGCTCCAGTGTAAT | 349 |
| Myb139 | ACTTCGTGATGAAAAGCTG | ATAGTTTTGTTGGCTGCTG | 159 |
| Myb13629 | GAGATTGAAAGCAGAAAAGG | CTGGAACAGGCATAGTTT | 152 |
| Myb10181 | CTGATTGCAGGGCGTATT | CTTCAACATCATTGCGTCC | 175 |
| Myb4344 | ATCCTGGAATGGCAATGT | GCAAAGCTCCAGTGTAAT | 313 |
| Myb4095 | GAGAAAACTTATCAGCATGG | AATATCCCTTTCCCTCAC | 323 |
| Myb19497 | GATTCTCTGTTACTTGCC | TGCTTCTCTCTATAGACC | 287 |
| Myb11378 | CTGATTGCAGGGCGTATT | CTTCAACATCATTGCGTCC | 172 |
| Myb4661 | GAAGACAGAGAAAAATGAGC | ACCAGCGAATCCATTTCA | 468 |
| Myb2894 | GAAGGCAGAGAAAAATGAAC | CTGGAACAGGCATAGTTT | 188 |
| Myb5330 | CTGATTGCAGGGCGTATT | CTTCAACATCATTGCGTCC | 172 |
| Myb4667 | GAAGGCAGAGAAAAATGAAC | GGAACAGGCATAGTTTTG | 186 |
| Myb2882 | GAGCTAAGCATCTTGTTGG | GTCTTGTTATTTGCTGCTTG | 321 |
| WRKY9928 | GCAAGAAAGCACGTAGAA | CATGGAGATTGCGCTAAA | 223 |
| WRKY8624 | ATGGAGGTGGCAGAAGAT | CCTCCATGAACACTTTTTTG | 365 |
| WRKY20368 | GAAGAAACAGACGAAGGA | CACCAAACTATCTTAAGCC | 246 |
| WRKY4666 | GCAGTATAACAACCAAAGAG | GATCCAAAAACTCCCCAAA | 138 |
| WRKY4214 | GCAAGAAAGCACGTAGAA | CATGGAGATTGCGCTAAA | 223 |
| WRKY2370 | ATGGAGGTGGCAGAAGAT | CCTCCATGAACACTTTTTG | 364 |
| WRKY16373 | TGCACCCATCCAAATTGT | ATTCCCAGTGTCATGTAG | 306 |
| WRKY10472 | TCACAACAAAGGAGCTTG | CCATTCTGGTCCTTGTCG | 214 |
| WRKY10455 | GATGTCAAAAAGAAGGATGC | TGTACAAATCCCACCTCC | 257 |
| WRKY5091 | AATAAAGGTGCAGAAGGC | CATCTCTTTCCTCCACCA | 294 |
| WRKY3145 | GGAGCAGTTGACCGAAGA | CCGCAGATATGAAACCATT | 305 |
| WRKY2211 | GGATAAGCTTACAGAGGA | AGATTTAGGAACTCGGCA | 210 |
| WRKY1289 | CACAACAAAAGAACTGGG | CACTTGGCCATCATAACT | 366 |
| MADS15369 | GGATATGGAACAAGGTGAA | CTCCTCAACAAAATCAAAGG | 236 |
| MADS6542 | TGGCACTATTCTGGGGAA | CTGATTGCCAACTCCTTT | 210 |
| MADS3510 | AGACACAGTGCTGGAAAA | CGGTCTGCAATCTCTTGT | 357 |
| MADS1818 | CCATCGCTGTTTTCCTTT | CTAACATTTCTCTTCCGGAT | 361 |
| MADS3172 | GGGAGCCTAAAAGTATGA | CAGCCCGCATTTCTTCTA | 207 |
| MADS20909 | CCCAGATGGAAAGACAATA | CCTCCATCAAATCTTTCTC | 269 |
| MADS2190 | ACCTCTGTATTTCCTCTG | CCATGAGCTACCTTCTTT | 332 |
| MADS2038 | GGGAGCCTAAAAGTATGA | CAGCCCGCATTTCTTCTA | 207 |
| MADS12384 | GGATATGGAACAAGGTGAA | CTCCTCAACAAAATCAAAGG | 236 |
| MADS3334 | GACAGAACAATGGAAAGG | CATCCATAAGTTGAACACC | 382 |
| MADS1317 | GTCATTGAAGAAGATAGTGG | GAGAGCAAAGCAACCAAA | 197 |
| MADS832 | CAGAGTGGCGGATGATTT | AGCCACAAGCACAAAAAG | 323 |
| PAL2688 | TTCGAATCTAAGTGGTGG | CCCTTTGTCATAACTTTCTC | 612 |
| CHS282 | GACAAGTCGGCAATAAAG | AGAATCTCCTCCGTCAAG | 50 |
| PAL1134 | TTTCAACAAGATTCCCGC | CAATATATTCGCCCGGAG | 217 |
| CHS1459 | GGACATGGTTGTGGTGGA | GATCTTCGACTTGGGCTG | 88 |
| F3'H2550 | GAAAGACGAGGTGAGCAA | GGTGAAGGGGATAAGAGAAA | 250 |
| F3H350 | GCGTGAGAGACGAAGTAA | GTGAAGGGGATAAGAGAAG | 254 |
| F3H483 | CAATGGCAAGTTCAAGAC | GGCAGACACCCATAAAAA | 332 |
| IGSTP346 | AGAAAATTCCGGTGCTCAT | CTTTCACTCCATCTGCCT | 462 |
| PAL1050 | CAACCAGGACGTCAATTC | AAAATCCGGTGTCGAGAA | 227 |
| IGSTP3726 | GAGACAACCTGATGCAAA | CAATCTCCCTCTCAAACT | 142 |
| IGSTP17883 | GTTCGCATAGCACTTTCT | CTCGGTCGTATGGATCTT | 226 |
| CHS381 | GACAAGTCGGCAATAAAG | TTGGTACATCATCACTCTC | 301 |
| CHS1053 | GCTAAGGCCATCAAGGAA | CACTGCAGACGACCAGAA | 243 |
| CHS2058 | GCGACAAGTCAGCAATAA | CAGCCGCTTCTTTTCCAA | 153 |
| CHS4014 | CGACAAGTCAGCAATAAAG | ACTGGTAGTGCAGAAAAC | 220 |
| CHS7594 | CATGGTTGTTGTGGAGGT | TTGGTACATCATCACTCTCT | 196 |
| IGSTP18952 | AGCTCATCACTTCTCGAAA | GTACTGGCCATCGTATTTT | 185 |
| IGSTP3638 | GAATATTGTGCAAGGAGAG | GTTCTGAAGAGGTTGTATG | 232 |
| IGSTP657 | TCTATGCGAGGAGTTTTC | TCGGGCATCACTTCTTTC | 302 |
| 3RT9058 | TGCAGAAGCCATAACGAA | AATTAGCATATCCCTCGC | 272 |
| IGSTP9956 | GAATCAGAGATAAGTGTGG | TAAGGATTGGTTTTGGGC | 312 |
| IGSTP9874 | GAGTACCTTGTGAATGCT | GTACTGGCCATCGTATTT | 216 |
| IGSTP985 | TTTTGATAATGGAGGTGCC | CAGTCAGACCCAACAAAT | 369 |
| IGSTP554 | CAGGTGGAAGAATGGGTG | GATCCGGCATAACTTCTTT | 259 |
| ALT14589 | GTTTAGTTCTCCTCGCTG | TTGCTTTTTGTGGTAGGTG | 474 |
| ALT14413 | TTGTGCTGGTAATTGTGG | TAATGCTTGGCTCTTGCT | 331 |
| ALT341930 | TATTTGGCGGCACTCTTG | GCCGGATTTGGTGGATTA | 514 |
| AST11138 | GAAGATTGGTTGGGCTAT | TTTGGGTCCATGTAGAAG | 351 |
| AST6108 | TCTCAGAAAGCAGGGGTT | CACTACCAGCATATCCCA | 49 |
| AST3968 | GACAGTTGCCTATAACAAAG | GGGCAAATACTCCTTATATC | 157 |
| AST3795 | TATGGCAGGTGTTACTAC | ATGAGAAACCAACTGGAC | 280 |
| AST1776 | GCTGTCCAAGCTCTATCT | GTCTCTGGATGGTAATAATGG | 172 |
| AST4 | GTCTACATGACAAAGGATG | GATGTCAGGTGCTTCAAA | 181 |
| NAD+3631 | CTCCAAATTGCAAGGTCT | CTTCCGTGCCTTGATAAT | 356 |
| AroE5840 | GATTGGGCGTTTGAACAG | ATTATCAACAAGAAGCGGCA | 142 |
| GPI1664 | TGTGGCCATTACTCAGGA | GTCCAACAGCTGACATTT | 122 |
| 6PGD3462 | GAGGCAATTGGTCGATGAT | GGCGGTAAGTGTCAAAATAGG | 355 |
| 6PGD2129 | GAATCTGAACTTTGGGGAA | TGTATGGAAAGAACCAGG | 346 |
| 6PGD1285 | GGGCTCTGTATGCATCTAA | TGGGCTTATTCTCTCTGG | 500 |
| RuBisCo4941 | CCAATTATGATGAGCGCC | CACATCTTCCCTTTCTTGA | 113 |
| RuBisCo1424 | TTGCTATAATGACAGGTGC | TCTAAGTTTTCGGTCCTC | 311 |
| RuBisCo459 | GCCATTGAGGAAGGTATT | TTCTGTAGAGCACACCTTG | 308 |
| diTP9764 | TTCGAAGGTGGTGGAAAT | TCGATGAACAGCTTGATG | 211 |
| diTP8780 | CCTTGTTAGAGGCAATTTTC | TTGCAAAGACTCGAAGGG | 325 |
| diTP977 | TCGAAGGTGGTGGAAATC | GTAAAAATGGCTCTACACG | 129 |
| AcI1998 | TACAAGAATGCAGTTCCC | GTAGCACCCTTAGCATTG | 158 |
| SUS4909 | TTGCAACGTGTAATGGAG | CCTTTCATAGATCCGCTG | 182 |
| SOD8804 | ATGTCCGACCAGATTATC | GTCAACAACCAAGGAGCA | 226 |
| SOD7730 | CATTGAGTGGACCTGATT | GCAACTCACTAATGCCAA | 303 |
| SOD6983 | GGGTAATTCACAAGTCGAG | TCCATCAGAACCAGCAAC | 250 |
| SOD2665 | CAGATTTTACAGAGCTCAAG | CTGTTGTATCCTTTTCGC | 621 |
| APX593 | TTGACAAAGCCAGGAGGA | AAACTAGGATCAGCAAGCA | 601 |
| ALT2898 | GGTCTGGATGTTGGGGAA | CAAAGTATCCTCCACGCT | 343 |
| AST3181 | CCAACTGGACCCCTAAAA | GTGTTACTACAGGCAATG | 264 |
| AST2487 | TCCCTTGACACGCTTAGA | TCTTTATATAGTCTGGCCAC | 197 |
| AST626 | TTAAAATTGGCACCGCACT | TTGGCACTATGATGTGGTT | 242 |
| NAD+4462 | CCTCTCTCACTGTTGATT | GCTCTAACAACATCCAAG | 416 |
| NAD+2599 | GTGCCTATGATTGCTAGA | ATCAACTCCATCTTCACC | 113 |
| AroE22855 | TGGGGAAGAGCATTAAGT | TAAGTGACAATTGCAGGG | 268 |
| 6PGD5962 | GTGACATACATTGGTAAAGG | TCCACAAGATACCCATCC | 248 |
| PFK6908 | GCGATGCTTTTGGACAGA | CCACTGACGAGCTTAAAT | 132 |
| RuBisCo2097 | GGACCGAAAACTTAGAATTG | TTCTGTAGAGCACACCTT | 354 |
| RuBisCo219 | TGAACTGGAGAGTGGAGAT | AATTTTGCTGGCTCTCCT | 39 |
| diTPS18523 | CAATGGAAGACGGTGAAAT | TCCAGGTTTGAAGTGCGA | 219 |
| diTPS11241 | CCTTGTTAGAGGCAATTTTC | AGTCAGCCATTGTCCATA | 79 |
| diTPS10990 | AATATACCCCCCATTGGA | GATGAGAGAGCATGAGTT | 115 |
| diTPS8769 | CTTGGTCCGGGCAGTATA | GAGGCGTTCAATTCAATCA | 180 |
| diTPS8495 | CGCCAAGGTGAAATAGTAA | CCATAAGTACCGCAGATT | 106 |
| diTPS5871 | TCTTCTGTTTCTTCGGTC | CCTCTTCCTTTAAACCTTTG | 195 |
| diTPS4679 | CCAAAAATGAACGATGAGC | CATCAGAGCAGGAAATACA | 69 |
| AcI15594 | TACAAGAATGCAGTTCCC | AATTGCACAGAAGACACC | 102 |
| SUS2581 | CTTTTACCCTTCCTGGTCT | AAAATCACCAGCAACCAC | 338 |
| SOD694 | GGAAAGTCGTGAATTGGAAG | GTCAACAACCAAGGAGCA | 196 |
| GR1894 | GTAGTTGAGGAGGGTTTG | TATCGGATTGGAAGACCT | 223 |
| APX858 | TTGACAAAGCCAGGAGGA | AAACTAGGATCAGCAAGCA | 600 |
| APX637 | GAAGCTTTCTGAATTGGG | GATTACAATATAGCGTGGG | 298 |
| ALT10459 | TTAGTTCTCCTCGCTGATG | ACAAAGATTCACAGATGCC | 246 |
| AST18254 | TTAAAATTGGCACCGCACT | TTGGCACTATGATGTGGTT | 242 |
| AST10567 | TCCCTTGACACGCTTAGA | CCACTTTTCAGGCCATTT | 181 |
| AST4142 | GTTTCTGCCTTCCAACAT | ACTTCCATCACCAAGAATCA | 270 |
| AST4000 | TGACAAAGGATGGGCGTA | GATGTCAGGTGCTTCAAAA | 174 |
| AST2456 | GGATAAGTATGGCAGGTC | TCAGTTCTTTGCCTCTTG | 179 |
| 6PGD3004 | GGGCTCTGTATGCATCTAA | TGGGCTTATTCTCTCTGG | 500 |
| 6PGD930 | GAATCTGAACTTTGGGGAA | TGTATGGAAAGAACCAGG | 346 |
| PFK7865 | GGTGAGGGATATGGTGAAA | CTCTGTTCTATATGCTGGT | 118 |
| PFK3336 | AATCCATCGCTCAACAATC | CCACTGACGAGCTTAAAT | 245 |
| RuBisCo14279 | TGAACTGGAGAGTGGAGAT | AATTTTGCTGGCTCTCCT | 39 |
| RuBisCo944 | TGAGGAAGGCATTGTTGT | AATAGAGCACACCCACCA | 39 |
| RuBisCo753 | GGACCGAAAACTTAGAATTG | TTCTGTAGAGCACACCTT | 354 |
| diTPS24918 | GTGAACAAGCTGCAAAGG | TGTTTCATGCAGAGATGGT | 112 |
| diTPS21613 | TTAGAGATGAGAATGGCGA | TCTCCCCGAATGTTCTTT | 216 |
| diTPS17530 | GGATTCGCAGAGCTCAAA | CATGCGCGTCATATAAGT | 157 |
| diTPS9193 | CACCCTCACTTTCCTCAA | GTAGCGAGTAGTCTGTCA | 107 |
| SUS11741 | GGAAATGTTTCTAGGAGG | GGCCAAAATAACCATGAG | 71 |
| SUS6064 | TTTCACCCTTCCTGGTCT | AATATCACCAGCAACCACA | 337 |
| SOD3240 | GAAAGTCGTGAATTGGAAGT | GGAGCGCATGTTCAAATTTA | 243 |
| SOD1764 | GGTAATTCACAAGTCGAG | TTCCATATTTTCCAGGGC | 454 |
| SOD1790 | CTCTTTCTGGGACACATT | CCAGCCTATAAATAACCAAC | 177 |
| GR3069 | AGCTAATGGTGATATCGG | GCAGTACCTTATCTTGGTT | 433 |
| APX8272 | GCCTCTCCAGTCTAATTAC | CAACTCATAAAAGGAACCTC | 164 |
| APX802 | TTTGGGGTTTGCAGTAGG | GTTAGAAGTGGATTACAGCG | 210 |
| APX345 | TTGACAAAGCCAGGAGGA | AAACTAGGATCAGCAAGCA | 601 |
| ALT4579 | CTGGATGTTGGGGAATTA | GTTGATAAACCTCGTCTG | 187 |
| ALT10393 | CTCGATTGACAACATTTCAC | CACTACCAACCTGCAAAC | 265 |
| AST6060 | CAGAAGAAGTTGGATGGT | AGAGACATCCGTTGCAATA | 117 |
| AST8832 | TCCCTTGACACGCTTAGA | CCACTTTTCAGGCCATTT | 181 |
| AST9313 | TTAAAATTGGCACCGCACT | TTGGCACTATGATGTGGTT | 242 |
| AST10035 | GTGTTACTACAGGCAATG | CCAACTGGACCCCTAAAA | 264 |
| AST12962 | GGATATGCTGGTAGTGAA | CTAAAAAGTCTCTCCTCTC | 67 |
| NAD+2715 | ACTGAAGTTGTGGAAGCAAA | GTGCACGGAGAGAAGATT | 94 |
| AroE16632 | ATTGGGCGTTTGAACAGA | ATTATCAACAAGAAGCGGCA | 142 |
| AroE1082 | GTTGATGCGGGAAATAGT | GAATGAGCACAAAAAAGTCC | 225 |
| 6PGD5950 | TATGGAGACATGCAGTTG | GCCATAATCATCCTTCACA | 168 |
| 6PGD4229 | GGAATCTGAACTTTGGGG | CGGCGGTAAGTATCAAAA | 253 |
| 6PGD2942 | GATATGTAGTTATGCGCAAG | AAACCAGGCATATCAGTC | 396 |
| 6PGD823 | GGAATCTGAACCTTGGGG | AATTCACCACTCTCCTCC | 182 |
| RuBisCo3648 | GAGGACCGAAAACTTAGA | CATAGCATTATACCCCAC | 282 |
| diTPS1535 | GGAGGTGGTGGAATTCAT | CTGGCTCAAACAAAAAAGAC | 99 |
| SOD9664 | GGAAAGTCGTGAATTGGAAG | GTCAACAACCAAGGAGCA | 196 |
| SOD5066 | TTGGTAGAGCACTTGTTG | CATATTTTCCAGGGCCTT | 144 |
| SOD4683 | GGCATCTCCTCTAACATT | TTGTGGGACCGTTATCTT | 215 |
| SOD551 | GATTTTACAGAGCTCAAGG | TCGAATGTGTCCCAGAAA | 390 |
| SOD4780 | GCTGGAACATATGACAAGAA | TTATCTGTCAACCCCATTC | 355 |
| SOD3685 | AAGGTCGAGATGTGTAAG | CGAGACGGAGCATGATTG | 127 |
| SOD74 | ACAGTTCCCCATAATCAC | AACTAGGATCAGCAAGCA | 389 |

Table S6. BLASTN search results against the NCBI database of the 22 candidate genes. As result are presented only the best BLAST hits with accession numbers of the sequences in the NCBI database.

| **Query sequence** | [**Max score**](https://blast.ncbi.nlm.nih.gov/Blast.cgi?CMD=Get&ALIGNMENTS=100&ALIGNMENT_VIEW=Pairwise&DATABASE_SORT=0&DESCRIPTIONS=100&DYNAMIC_FORMAT=on&FIRST_QUERY_NUM=0&FORMAT_OBJECT=Alignment&FORMAT_PAGE_TARGET=&FORMAT_TYPE=HTML&GET_SEQUENCE=yes&I_THRESH=&LINE_LENGTH=60&MASK_CHAR=2&MASK_COLOR=1&NUM_OVERVIEW=100&PAGE=MegaBlast&QUERY_INDEX=0&QUERY_NUMBER=0&RESULTS_PAGE_TARGET=&RID=1ZK2CUWD015&SHOW_LINKOUT=yes&SHOW_OVERVIEW=yes&STEP_NUMBER=&OLD_VIEW=false&DISPLAY_SORT=1&HSP_SORT=1) | **Total score** | **Query cover** | **E-value** | **Ident** | **Accession number in NCBI** |
| --- | --- | --- | --- | --- | --- | --- |
| Myb 4633 | 459 | 459 | 99% | 3e-125 | 95% | BT070763.1 |
| Myb 4095 | 337 | 337 | 72% | 1e-88 | 96% | FJ125922.1 |
| WRKY9928 | 278 | 278 | 85% | 4e-71 | 100% | EU393816.1 |
| WRKY20348 | 305 | 305 | 100% | 2e-79 | 94% | EF676996.1 |
| WRKY4214 | 281 | 281 | 85% | 3e-72 | 100% | EU393816.1 |
| WRKY1289 | 510 | 510 | 100% | 8e-141 | 97% | BT070825.1 |
| MADS15369 | 241 | 228 | 100% | 4e-56 | 92% | BT119355.1 |
| MADS1818 | 459 | 459 | 99% | 3e-125 | 93% | BT104866.1 |
| MADS2038 | 215 | 215 | 100% | 3e-52 | 94% | BT117271.1 |
| MADS12384 | 251 | 261 | 97% | 5e-66 | 92% | BT119355.1 |
| PAL1134 | 294 | 294 | 100% | 4e-76 | 97% | AY321089.1 |
| CHS381 | 298 | 298 | 100% | 4e-77 | 91% | JN400054.1 |
| CHS4014 | 237 | 237 | 100% | 6e-59 | 97% | KJ796482.1 |
| F3H350 | 363 | 363 | 100% | 1e-96 | 99% | KF704818.1 |
| IGSTP657 | 363 | 363 | 98% | 2e-96 | 93% | JX962799.1 |
| diTPS11241 | 235 | 235 | 100% | 2e-58 | 99% | KJ158966.1 |
| diTPS5871 | 237 | 237 | 100% | 5e-59 | 98% | GU045757.1 |
| APX802 | 217 | 217 | 57% | 2e-52 | 93% | EF677796.1 |
| APX8272 | no significant similarity found | | | | | |
| APX637 | no significant similarity found | | | | | |
| SOD4683 | no significant similarity found | | | | | |
| SOD3685 | 111 |  | 111 | 1e-21 | 98% | FN564373.1 |

**Figures**


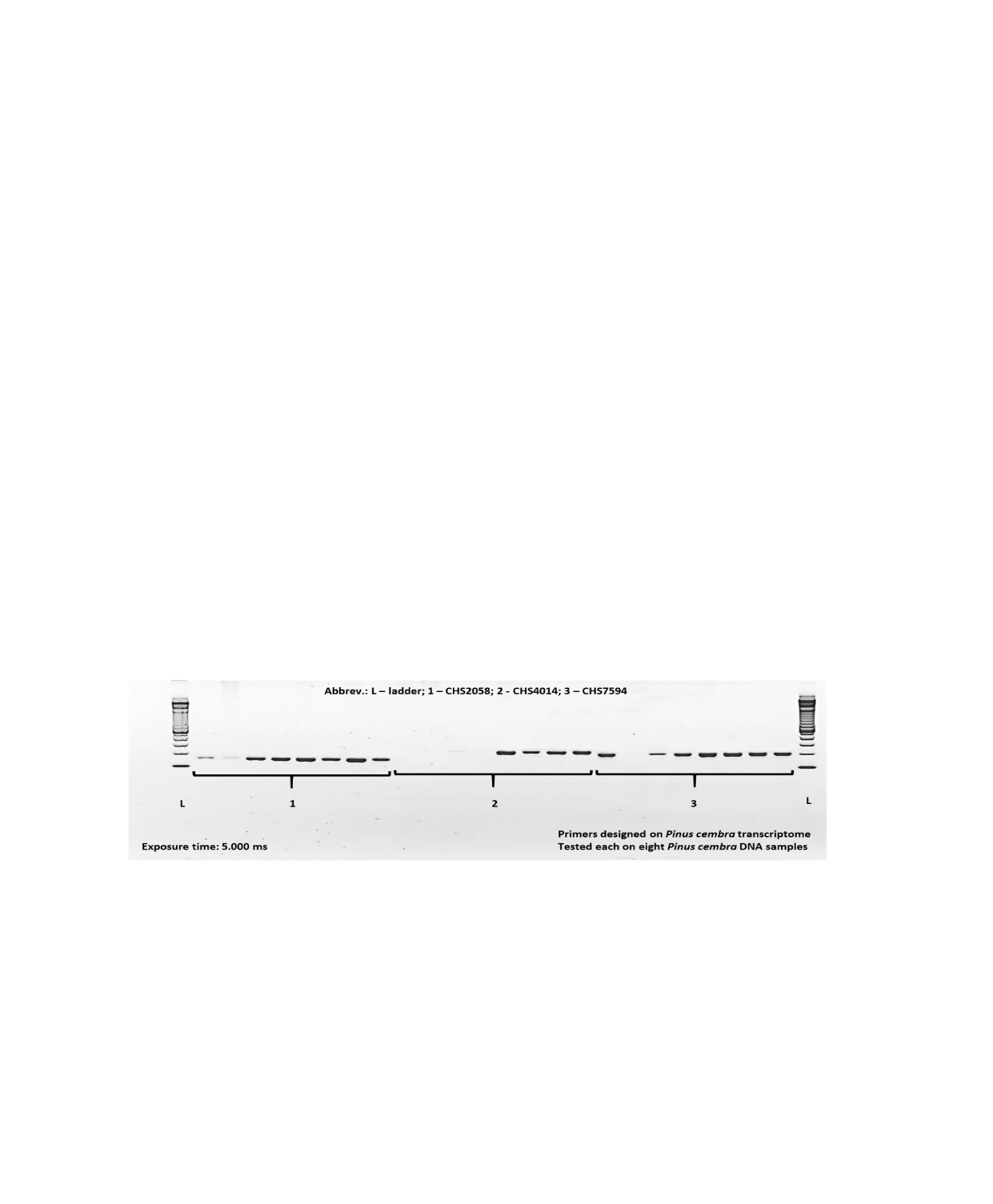


Fig S1. *Pinus cembra* PCR products amplified by three selected primers (CHS2058, CHS4014, CHS7594) analyzed on 2% agarose gel.


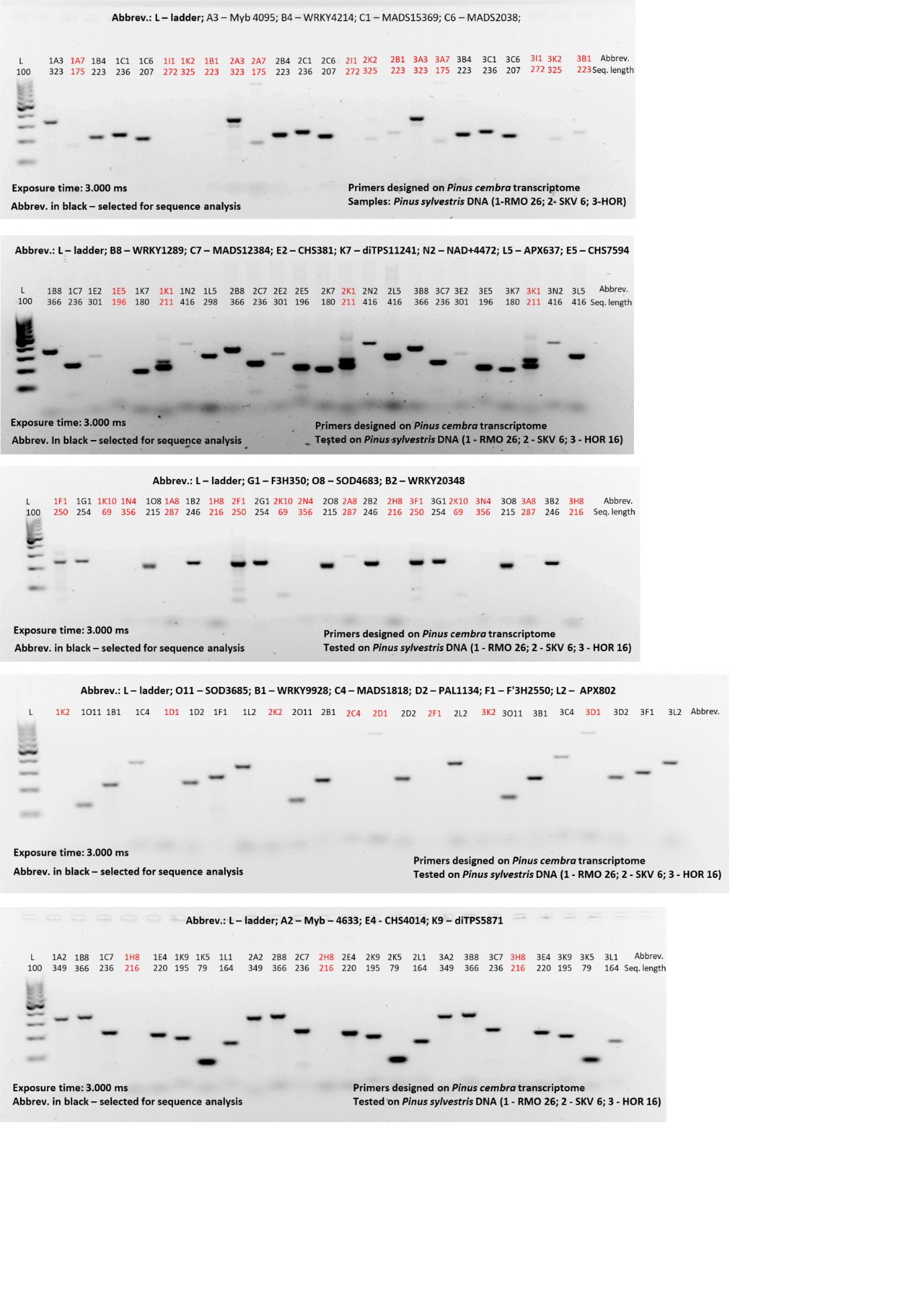


Fig S2. PCR products of *Pinus sylvestris* with the selected primers analyzed on 2% agarose gels. Sequences abbreviated in black were selected for sequence analysis. The number under each abbreviation indicates the length of the sequence.

**References**

1. Tao DL, Oquist G, Wingsle G (1998) Active oxygen scavengers during cold acclimation of Scots pine seedlings in relation to freezing tolerance. Cryobiology 37:38–45. <https://doi.org/10.1006/cryo.1998.2096>
2. Anderson JV, Chevone B, Hess JL (1992) Seasonal variation in the antioxidant system of eastern white pine needles. Plant Physiol 98:501–508. <https://doi.org/10.1104/pp.98.2.501>
3. Tang W (2000) Peroxidase activity of desiccation-tolerant loblolly pine somatic embryos. In Vitro Cell Dev-Pl, 36:488–491. <https://doi.org/10.1007/s11627-000-0087-2>
4. Whittet R, Cavers S, Cottrell J, Rosique-Esplugas C, Ennos R (2017) Substantial variation in the timing of pollen production reduces reproductive synchrony between distant populations of *Pinus sylvestris* L. in Scotland. Ecol Evol 7:5754–5765. <https://doi.org/10.1002/ece3.3154>
5. Yang Y, Han C, Liu Q, Lin B, Wang J (2008) Effect of drought and low light on growth and enzymatic antioxidant system of *Picea asperata* seedlings. Acta Physiol Plant 30:433–440. <https://doi.org/10.1007/s11738-008-0140-z>
6. Yamazaki JY, Ohashi A, Hashimoto Y, Negishi E, Kumagai S, Kubo T, Kamimura Y (2003) Effects of high light and low temperature during harsh winter on needle photodamage of *Abies mariesii* growing at the forest limit on Mt. Norikura in Central Japan. Plant Sci 165:257–264. <https://doi.org/10.1016/S0168-9452(03)00169-9>
7. Polle A, Kroniger W, Rennenberg H (1996) Seasonal fluctuations of ascorbate-related enzymes: Acute and delayed effects of late frost in spring on antioxidative systems in needles of Norway spruce (*Picea abies* L). Plant Cell Physiol 37:717–725. <https://doi.org/10.1093/oxfordjournals.pcp.a029005>
8. Pukacka S and Pukacki M (2000) Seasonal changes in antioxidant level of scots pine (*Pinus sylvestris* L.) needles exposed to industrial pollution. I. Ascorbate and thiol content. Acta Physiol Plant 22:451–456. <https://doi.org/10.1007/s11738-000-0088-0>
9. Schröder P, Rennenberg H (1992) Characterization of glutathione S-transferase from dwarf pine needles (*Pinus mugo* Turra). Tree Physiol 11:151–60. <http://www.ncbi.nlm.nih.gov/pubmed/14969958>
10. Messner B, Boll M, Berndt J (1991) L-Phenylalanine ammonia-lyase in suspension culture cells of spruce (*Picea abies*). Plant Cell Tiss Org 27:267–274. <https://doi.org/10.1007/bf00157590>
11. Teklemariani TA, Blake TJ (2004) Phenylalanine ammonia-lyase-induced freezing tolerance in jack pine (*Pinus banksiana*) seedlings treated with low, ambient levels of ultraviolet-B radiation. Physiol Plantarum 122:244–253. <https://doi.org/10.1111/j.0031-9317.2004.00396.x>
12. El-Kassaby ZA (1981) Genetic interpretation of malate dehydrogenase isozymes in some conifer species. J Hered 72:451–452. <https://doi.org/10.1093/oxfordjournals.jhered.a109560>
13. Polle A, Rennenberg H (1991) Superoxide dismutase activity in needles of Scots pine and Norway spruce under field and chamber conditions: lack of ozone effects. New Phytol 117:335–343. <https://doi.org/10.1111/j.1469-8137.1991.tb04915.x>
14. Karpinski, S., Wingsle, G., Karpinska, B., & Hallgren, J. E. (1993) Molecular responses to photooxidative stress in *Pinus sylvestris* (L.)(II. differential expression of CuZn-superoxide dismutases and glutathione reductase. Plant Physiol 103:1385-1391.
15. Miller B, Madilao LL, Ralph S, Bohlmann J (2005) Insect-induced conifer defense. White pine weevil and methyl jasmonate induce traumatic resinosis, de novo formed volatile emissions, and accumulation of terpenoid synthase and putative octadecanoid pathway transcripts in Sitka spruce. Plant Physiol 137:369–382. <https://doi.org/10.1104/pp.104.050187>
16. Johnson MA, Croteau R (1987) Biochemistry of conifer resistance to bark beetles and their fungal symbionts. Ecology and metabolism of plant lipids. ACS Symp Ser 325:76–91. <https://doi.org/doi:10.1021/bk-1987-0325.ch006>
17. Chen F, Zhang X, Liu X, Zhang L (2017) Evolutionary analysis of MIKCc-type MADS-box genes in gymnosperms and angiosperms. Front Plant Sci 8:1–11. <https://doi.org/10.3389/fpls.2017.00895>
18. Mouradov A, Glassick T, Teasdale R (1997) Isolation and characterization of a new MADS-box cDNA from *Pinus radiata*. Plant Physiol 113:96–002.
19. Mouradov A, Glassick T, Vivian-Smith A, Teasdale R (1996) Isolation of a MADS box gene family from *Pinus radiata*. Plant Physiol 110:1047.
20. Rutledge R, Regan S, Nicolas O, Fobert P, Côté C, Bosnich W, Stewart D (1998) Characterization of an AGAMOUS homologue from the conifer black spruce (*Picea mariana*) that produces floral homeotic conversions when expressed in *Arabidopsis*. Plant J 15:625–634. <https://doi.org/10.1046/j.1365-313X.1998.00250.x>
21. Slater G, Birney E, Box G, Smith T, Waterman M, Altschul S, Salzberg S (2005) Automated generation of heuristics for biological sequence comparison. BMC Bioinformatics 6:31. <https://doi.org/10.1186/1471-2105-6-31>
22. Sundström J, Carlsbecker A, Svensson ME, Svenson M, Johanson U, Theißen G, Engström P (1999) MADS-box genes active in developing pollen cones of Norway spruce (*Picea abies*) are homologous to the B-class floral homeotic genes in angiosperms. Dev Genet 25:253–266. [https://doi.org/10.1002/(SICI)1520-6408(1999)25:3<253:AID-DVG8>3.0.CO;2-P](https://doi.org/10.1002/(SICI)1520-6408(1999)25:3%3c253:AID-DVG8%3e3.0.CO;2-P)
23. Tandre K, Albert VA, Sundås A, Engström P (1995) Conifer homologues to genes that control floral development in angiosperms. Plant Mol Biol 27:69–78. <https://doi.org/10.1007/BF00019179>
24. Bedon F, Bomal C, Caron S, Levasseur C, Boyle B, Mansfield SD, MacKay J (2010) Subgroup 4 R2R3-MYBs in conifer trees: Gene family expansion and contribution to the isoprenoid-and flavonoid-oriented responses. J Exp Bot 6:3847–3864. <https://doi.org/10.1093/jxb/erq196>
25. Bedon F, Grima-Pettenati J, Mackay J (2007) Conifer R2R3-MYB transcription factors: sequence analyses and gene expression in wood-forming tissues of white spruce (*Picea glauca*). BMC Plant Biol 7:17 <https://doi.org/10.1186/1471-2229-7-17>
26. Xue B, Charest PJ, Devantier Y, Rutledge RG (2003) Characterization of a MYBR2R3 gene from black spruce (*Picea mariana*) that shares functional conservation with maize C1. Mol Genet Genomics 270 (1):78–86. <https://doi.org/10.1007/s00438-003-0898-z>
27. Osakabe Y, Osakabe K, Chiang VL (2009) Characterization of the tissue-specific expression of phenylalanine ammonia-lyase gene promoter from loblolly pine (*Pinus taeda*) in *Nicotiana tabacum*. Plant Cell Rep 28:1309–1317. <https://doi.org/10.1007/s00299-009-0707-1>
28. Ersoz ES, Wright MH, González-Martínez SC, Langley CH, Neale DB (2010) Evolution of disease response genes in loblolly pine: Insights from candidate genes. PloS one 5:e14234. <https://doi.org/10.1371/journal.pone.0014234>
29. Donini P, Liu J-J, Ekramoddoullah AKM (2009) Identification and characterization of the WRKY transcription factor family in *Pinus monticola*. Genome 52:77–88. <https://doi.org/10.1139/G08-106>
